# Structural biology of complement receptors

**DOI:** 10.3389/fimmu.2023.1239146

**Published:** 2023-09-11

**Authors:** Jorge Santos-López, Karla de la Paz, Francisco J. Fernández, M. Cristina Vega

**Affiliations:** ^1^ Centro de Investigaciones Biológicas Margarita Salas, Consejo Superior de Investigaciones Científicas (CSIC), Madrid, Spain; ^2^ Research & Development, Abvance Biotech SL, Madrid, Spain

**Keywords:** complement, complement receptors, structural biology, CR1/CR2, CR3/CR4, CRIg, C5aR1/C5L2/C3aR, host-pathogen interactions

## Abstract

The complement system plays crucial roles in a wide breadth of immune and inflammatory processes and is frequently cited as an etiological or aggravating factor in many human diseases, from asthma to cancer. Complement receptors encompass at least eight proteins from four structural classes, orchestrating complement-mediated humoral and cellular effector responses and coordinating the complex cross-talk between innate and adaptive immunity. The progressive increase in understanding of the structural features of the main complement factors, activated proteolytic fragments, and their assemblies have spurred a renewed interest in deciphering their receptor complexes. In this review, we describe what is currently known about the structural biology of the complement receptors and their complexes with natural agonists and pharmacological antagonists. We highlight the fundamental concepts and the gray areas where issues and problems have been identified, including current research gaps. We seek to offer guidance into the structural biology of the complement system as structural information underlies fundamental and therapeutic research endeavors. Finally, we also indicate what we believe are potential developments in the field.

## 1 Introduction to the complement receptors

The currently accepted list of complement receptors includes four broad structural classes of transmembrane proteins: complement control protein (CCP)/short consensus repeat (SCR) domain modular single-pass transmembrane receptors (CR1, CR2), β_2_ integrins (CR3, CR4), complement receptor of the immunoglobulin family (CRIg), and G-protein coupled receptors (GPCR) (C5aR1, C5aR2, C3aR) (**Table 1**). Various aspects of their sequence, function, structure, localization, regulation, activation, and implications for infection, pathogenesis, and therapy have been reviewed elsewhere (1–19). Complement receptors recognize either the C3 proteolytically activated fragments C3b, iC3b, and C3dg deposited on opsonized surfaces (CR1 to CR4 and CRIg) or the soluble anaphylatoxins released during alternative pathway activation (C3aR) or terminal pathway activation (C5aR1/C5aR2). The pattern of cellular expression and compartmentalization of the complement receptors underlies their various functions: leukocyte recruitment and migration, phagocytosis, and inflammation. A fascinating function of complement receptors is connecting the effector responses of the innate and adaptive branches of the immune system.

**Table 1:** Complement receptors and membrane-associated regulators.

Complement receptor^1^	Structural class	Ligands^2^	PDB ID^3^
**CR1** CD35	Single-pass (bitopic) membrane protein CCP/SCR mosaic protein 30 CCP domains (~220 kDa) Other spliced isoforms contain 23, 37, or 44 CCP domains	C3b / C4b AP / CP C3 convertase C5 convertase Other ligands: C1q, MBL, and iC3b/C3d(g) with low affinity	sCR1 (SAXS: 2Q7Z) CCPs 1-2 (NMR: 2MCZ) CCPs 2-3 (NMR: 2MCY) CCP16 (NMR: 1PPQ) CCPs 16-17 (NMR: 1GKG) CCPs 15-17 (NMR: 1GKN) CCPs 15-17:C3b (XRD: 5FO9)
**CR2** CD21	Single-pass (bitopic) membrane protein CCP/SCR mosaic protein 15 CCP domains (~145 kDa)	iC3b C3d(g) Other ligands: IFNα, Low-affinity IgE receptor CD23	sCR2 (SAXS: 2GSX) CCPs 1-2 (XRD: 1LY2; NMR: 1W2R) CCPs 1-2:C3d (XRD: 1W2S, 3OED)
**CR3** ^4^ CD11b+CD18 α_M_β_2_ Mac-1	Heterodimer of single-pass (bitopic) subunits Integrin superfamily 170 kDa (α_M_) + 95 kDa (β_2_)	iC3b C3d(g) C3(H_2_O) Other ligands: ICAMs, Fibrinogen, Plasminogen, LPS (many others)	α_M_I (XRD: 1BHO, 1BHQ, 1IDN, 1IDO, 1JLM, 1M1U, 1MF7, 1N9Z, 1NA5) Cytoplasmic domain (NMR: 2LKE, 2LKJ) CR3 headpiece (XRD: 7P2D) CR3 ectodomain (cEM: 7USM) α_M_I:C3d (XRD: 4M76) α_M_I:iC3b (XRD: 7AKK)
**CR4** ^4^ CD11c+CD18 α_X_β_2_ p150/95	Heterodimer of single-pass (bitopic) subunits Integrin superfamily 150 kDa (α_X_) + 95 kDa (β_2_)	iC3b Other ligands: ICAM-1, VCAM-1, Fibrinogen, LPS, Heparin (others)	α_X_I (XRD: 1N3Y) Cytoplasmic domain (NMR: 2LUV) CR4 ectodomain (closed) (XRD: 3K71, 3K72, 3K6S, 5ES4) CR4 ectodomain (metastable) (XRD: 4NEN, 4NEH)
Only CD18^4^			I-EGF2-3 (NMR: 1LY3) PSI/Hybrid/I-EGF1 (XRD: 1YUK, 5E6V) + I-EGF2 (XRD: 2P26, 5E6W) + I-EGF3 (XRD: 2P28, 5E6X) β_2_ TM helix (NMR: 5ZAZ)
**CRIg** VSIG4 Z39Ig	Single-pass (bitopic) membrane protein Ig-like superfamily ~42 kDa	C3b iC3b	V-set Ig-like 1 (XRD: 2ICC) V-set Ig-like 1:C3c (XRD: 2ICE) V-set Ig-like 1:C3b (XRD: 2ICF) CRIg (AlphaFold AF-Q9Y279-F1)
**C5aR1** CD88	G-protein coupled receptor ~39 kDa	C5a C5a^desArg^ Other ligands: C3a, ribosomal protein S19	C5aR1:NDT9513727 (XRD: 5O9H) C5aR1:PMX53:NDT9513727 (XRD: 6C1Q) C5aR1:PMX53:Avacopan (XRD: 6C1R) C5aR1:G_i_:C5a (cEM: 7Y64) C5aR1:G_i_:C5a^pep^ (cEM: 7Y65) C5aR1:G_i_:BM213 (cEM: 7Y66) C5aR1(I116A):G_i_:C089 (cEM: 7Y67)
**C5aR2** C5L2 GPR77	Class A (Rhodopsin) G-protein coupled receptor 36 ~kDa	C5a C5a^desArg^	No structure available
**C3aR** C3aR1	Class A (Rhodopsin) G-protein coupled receptor ~53 kDa	C3a Other ligands: C5a	C3aR:G_i_ (cEM: 8HK3) C3aR:G_i_:C3a (cEM: 8HK2) C3aR:G_i_:C5a (cEM: 8HK5)

^1^ The name of the complement receptor used in this review appears in bold. Alternative names and CD nomenclature (if available) are also indicated.

^2^ Main (canonical) ligands are given first. Other ligands are listed, although no attempt has been made to classify them according to their physiological relevance. Viral proteins that hijack complement receptors to gain entry to the target cell have not been included.

^3^ The list of PDB entries is not meant to be exhaustive. In choosing among available structures, we have placed the emphasis in those that have contributed crucial information to the understanding of the architecture of the receptors. Accordingly, we have omitted a few structures featuring only short peptides derived from complement receptors (e.g., for CR4) or when they represent antibody/small-molecule complexes that do not significantly alter our structural understanding of the receptor (e.g., CR3 α_M_I).

^4^ For the integrin receptors (CR3/CR4), we have arranged the PDB IDs of structures containing CD11b/α_M_ chain sequences under CR3, those containing CD11c/α_X_ chain sequences under CR4, and those containing exclusively CD18/β_2_ chain sequences under “Only CD18”.

The membrane-bound negative complement regulators membrane cofactor protein (MCP/CD46) and decay accelerating factor (DAF/CD55) are structurally related to CR1 and CR2 as they contain tandem repetitions of the CCP/SCR domain (20). However, they are not commonly considered complement receptors as their main function is to accelerate convertase decay or act as cofactor of complement factor I (FI) to prevent unregulated complement deposition on self-cell surfaces, and will not be discussed further (but see (21, 22) regarding MCP as a complement receptor in T-cell lymphocytes). Likewise, we will not cover three proteins that have been proposed as C1q receptors: C1qRp/CD93, cC1qR/calreticulin (CR), and gC1qbp. CD93, also known as C1q receptor protein (C1qRp), promotes cell adhesion, migration, and angiogenesis; however, its main ligand appears to be multimerin-2, not C1q (23, 24). cC1qR is a 62-kDa form of ecto-calreticulin that can bind the collagen domain in C1q with the assistance of other membrane proteins including β_1_ integrins, CD91, and the KDEL docking receptor (25). Finally, gC1qR is a 33-kDa binding protein for the globular head of C1q (gC1qbp) found in both the intracellular and cell surface compartments, where it can bind various proteins including vitronectin, thrombin, and fibrinogen; the location of gC1qR is mainly in the mitochondria, where its leader peptide is processed (24).

The renewed interest in the pharmacological modulation of the complement system (26–31) has contributed to a recent surge of structural information on the structure and function of complement receptors, especially CR3 (32–34) and the anaphylatoxin receptors C5aR1 and C3aR (35, 36). This review aims to inform and guide structurally aware basic and clinical research by providing an up-to-date synthesis of our current understanding of the structural biology of complement receptors.

An exciting topic that we will not cover in this review is the intracellular complement system, the complosome, despite the breadth of attributed effector functions and the important associations uncovered with prevalent diseases (reviewed in (37)). We have decided to leave the complosome outside the scope of this review as the available structural information is not specific to the intracellular location of complosome components (e.g., C3aR/C5aR1).

## 2 Highly modular complement receptors based on the CCP/SCR domain

### 2.1 The building block: CCP/SCR domain

The complement receptors CR1 and CR2 and the negative complement regulators FH, FH-related (FHR) proteins, C4b binding protein (C4BP), MCP, and DAF are all mosaic proteins comprised of several independently-folded modules known as the short consensus repeat (SCR), complement control protein (CCP), or Sushi domain (20, 38). Other complement factors that bind C3b or C4b, like FB or FI, also contain CCP domains and other structural and catalytic domains. Conversely, CCP domains are also found in complement factors lacking the ability to bind C3b or C3b, like the complement proteases C1r and C1s (39).

**Figure 1:**
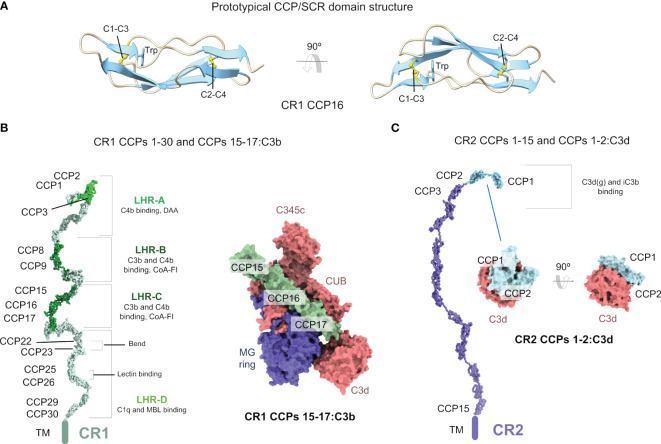
Complement receptors CR1 and CR2 are mosaic proteins built from CCP/SCR modules. **(A)** Structure of a prototypical CCP/SCR domain, the CR1 CCP16, taken from the structure of CR1 CCPs 15-17:C3b (PDB ID 5FO9). The domain is shown in cartoons in two orientations. The two most conserved features of CCP domains, the disulfide bonds between four conserved cysteine residues (C1-C3 and C2-C4) and a conserved tryptophan (W) residue, are shown in sticks and CPK atom colors. **(B)** Structure of the CR1 ectodomain (left) comprising CCPs 1-30 modeled from SAXS data (PDB ID 2Q7Z), with C3b/C4b interacting CCP domains colored in lime (C4b) and dark green (C3b/C4b). Structure of the CR1 CCPs 15-17:C3b (right) (PDB ID 5FOB) in molecular surface representation. C3b is colored according to chain (the α’ chain in red, the β chain in blue), and CR1 CCPs 15-17 is colored in dark green. **(C)** Structure of the CR2 ectodomain (left) comprising CCPs 1-15 modeled from SAXS data (PDB ID 2GSX), with C3b interacting CCP domains colored in cyan. Structure of the CR2 CCPs 1-2:C3d (right) (PDB ID 3OED) in molecular surface representation. C3d is colored in red, and CR2 CCPs 1-2 is colored in cyan.

The dimensions of the CCP domain are approximately 1 nm in width and 3.6 nm in length (**Figure 1A**). Each CCP (61 amino acids) contains a hydrophobic core composed of up to eight-stranded antiparallel β-sheet stabilized by two conserved disulfide bridges (Cys1-Cys3 and Cys2-Cys4) and a buried conserved Trp residue (40) (**Figure 1A**). Individual CCP domains in mosaic sequences start with the first conserved Cys (Cys1) and end with the last conserved Cys (Cys4); the two-to-eight residues between Cys4 in the preceding CCP domain and Cys1 in the following CCP domain are denoted as inter-CCP linking regions. A structurally important feature of the inter-CCP linkers is that they allow a wide range of inter-domain orientations, thus adding to the structural variability of the mosaic proteins that contain them (41). CCP domains have been structurally classified into nine distinct groups (from A to I) according to sequence and structural features (42). This classification is intended to help the systematic structural analysis and the accurate homology modeling of CCP domain-containing proteins.

### 2.2 Complement receptor 1

Complement receptor 1 (CR1, CD35, C3b/C4b receptor) is a type I transmembrane glycoprotein from the Regulators of Complement Activity (RCA) family. CR1 acts as a receptor for C3b/C4b, C1q (43), the mannan-binding lectin (MBL) (44), and as an immune adherence receptor (45). The biological functions of CR1 rely on its ability to bind to C3b and C4b reversibly, components of the C3 convertase of the alternative pathway (C3bBb) or the classical pathway (C4b2a), inactivating the C3 and C5 convertases, and promoting the dissociation of the catalytic subunits C3a or Bb (decay-accelerating activity) (46). CR1 also serves as a necessary cofactor for FI-mediated proteolytic cleavage of C3b and C4b to the breakdown products iC3b/C3dg and iC4b/C4dg, respectively (cofactor activity) (46). The cellular location of CR1 influences its biological functions. CR1 is mainly found on the surface of erythrocytes, where it is responsible for the Knops blood group (York and McCoy antigens), and on antigen-presenting (APC) cells (47). While in erythrocytes CR1 contributes to the clearance of complement-fixed immune complexes, in leukocytes its main role seems to be channeling the immune response to foreign antigens to other immune cell types bearing CR2, CR3, and CR4 receptors. In addition, CR1 acts as a B-cell receptor (BCR) inhibitor to prevent B cell activation (48). A soluble version of CR1 (sCR1) has also been identified with anti-inflammatory properties (43).

#### 2.2.1 Structure of the largest complement receptor, CR1

CR1 is the largest member of the RCA family. In the most common allelic form, the extra-cellular component of CR1 (sCR1) contains 30 CCP domains and 14 occupied *N*-linked glycosylation sites; other allelic forms have 23, 37, or 44 CCP domains. The 30 CCP domains of CR1 are organized as four long homologous repeat regions spanning seven CCP domains (LHR-A to LHR-D) plus two C-terminal CCP domains. Three functionally relevant sites have been identified in CR1: Site 1 in CCPs 1-3 in LHR-A binds C4b and is the site harboring the decay-accelerating activity toward the classical and alternative pathway C3 convertases; site 2 in CCPs 8-10 in LHR-B and site 3 in CCPs 15-17 in LHR-C bind C3b and C4b and are the main sites for the FI cofactor activity.

Given the membrane location, size, glycosylation, and highly modular and flexible structure, obtaining high-resolution structural information about CR1 has been challenging. The first structural information about sCR1 was obtained by negative staining electron microscopy (NS-EM). Those early electron micrographs exposed the elongated structure of sCR1 in various CCP structural arrangements (49). However, the most comprehensive structural description of sCR1 has thus far been obtained by solution small-angle X-ray scattering (SAXS) coupled with biophysical techniques like analytical ultracentrifugation (AUC) and constrained computational modeling.

Even though at a moderate resolution, this approach has revealed critical structural features of CCP repeat proteins like FH (50), CR2 (51), and CR1 (52). Strikingly, sCR1, as FH and sCR2, does not adopt a fully extended structure, which would stick out of the cellular surfaces harboring it by a maximum theoretical length of 108 nm (compared with 17 nm for C3b/C4b). In contrast, SAXS data analysis revealed that sCR1 structure folds back onto itself to yield a more densely packed molecule with a maximum dimension *D*_max_ = 55 nm and a radius of gyration *R*_G_ = 13.4 nm (52) (PDB ID 2Q7Z) (**Figure 1B**). In comparison, FH has a *D*_max_ = 40 nm instead of the maximum theoretical length of 73 nm, and sCR2 has a *D*_max_ = 38 nm instead of the theoretically maximum length of 54 nm. Analysis of the SAXS cross-sectional radii of gyration for sCR1 (*R*_XS-1 _= 4.7 nm and *R*_XS-2 _= 1.2 nm) in combination with the *R*_G_ and the frictional ratio *R*_G_/*R*_0 _= 3.76 provided further confirmation of the folded-back structure of sCR1, which resembles that of FH more closely than that of sCR2. For CCP-containing proteins, *R*_XS-1_ reports on the averaged medium-range folding back and *R*_XS-2_ on the averaged short-range inter-CCP orientation between adjacent CCP domains (50). Equilibrium sedimentation experiments with sCR1 resulted in a sedimentation coefficient *s*°_20,w_ = 5.84, and a frictional coefficient anisotropy ratio *f/f*_0 _= 2.3 (compared to 1.25 for globular proteins), which agree with the extended but folded-back structural model derived from SAXS measurements.

SAXS-constrained modeling of the three-dimensional structure of sCR1 has shown that sCR1 can be best described as a family of partly folded-back CCP structural arrangements with a moderate degree of flexibility around the CCP inter-linking regions. SAXS and other biophysical techniques have comprehensively sampled the translational and rotational average structural ensemble. SAXS-constrained modeling has suggested that the four-residue linker between CCP22 and CCP23 might be responsible for the main bend in sCR1. Still, this inference awaits experimental corroboration as SAXS alone cannot prove where the kink occurs. A kink at CCP22-CCP23 would introduce an angle between the LHR-C and LHR-D regions, apparently without implications for ligand recognition, as the C3b/C4b binding sites are in the LHR-A, LHR-B, and LHR-C regions. This observation contrasts with the oft-cited statement that the folded-back structure of CR1 facilitates binding to multiple ligands on the surface of pathogens or immune complexes.

Higher resolution structures of unliganded CR1 domains are only available for a subset of CCP domains. The earliest structures were determined by multinuclear NMR for the three CCP domain tandem constructs CCPs 1-3, CCPs 8-10, and CCPs 22-24 (41), and the CCPs 15-16 (PDB ID 1GKN) and CCPs 16-17 (PDB ID 1GKG) domain pairs in site 3 of CR1 (53). The NMR structure of an individual CCP domain was also published for CCP16 (PDB ID 1PPQ) (54). The NMR structure of the N-terminal CCP domain pairs CCPs 1-2 (PDB ID 2MCZ) and CCPs 2-3 (PDB ID 2MCY) (55).

#### 2.2.2 CR1 dampens C3 and C5 convertase activities, and transports opsonized cargo

CR1 uses CCP binding sites in LHR-A, LHR-B, and LHR-C to recognize and bind C3b and C4b. CR1 ligands include both monomeric ligands such as C3b, C4b, or the C3 convertase (C3bBb or C4b2a) and the bivalent C5 convertases, which contain a back-to-back arrangement of either C3b-C3b dimers (alternative pathway) or C3b-C4b dimers (classical pathway) (56, 57). This bivalent recognition sets CR1 apart from all other RCA proteins and it explains the 10-fold tighter binding affinity of CR1 for C5 convertases over C3 convertases (58).

Recent structural data have shed light on how CR1 recognizes its cognate ligand C3b (59). The crystallographic structure of C3b:CR1 CCPs 15-17 (PDB ID 5FO9) has shown how CR1 exploits a similar binding mode to FH CCPs 1-4 to exert cofactor activity (60) (**Figure 1C**). C3b domains engaged in this interaction include MG7, MG6, CUB, MG2, MG1, and TED, and the α’NT region over a region spanning ~100 Å with ~1910 Å^2^ buried surface area.

In contrast to C3b/C4b ligands, CR1 uses the LHR-D homologous repeat to recognize and bind C1q and MBL. Therefore, C1q and MBL must compete for binding to CR1. A structural characterization of the interaction between CR1 and C1q/MCP awaits further investigation.

#### 2.2.3 Implications of CR1 structure for disease and immune evasion

sCR1 has been proposed for therapeutic use based on its anti-inflammatory properties and low immunogenicity. Possible applications include controlling inflammatory tissue damage in myocardial infarction (49), tissue damage suppression in complement-dependent autoimmune diseases (46), and the treatment of pemphigus foliaceus (61).

CR1 is also known as a receptor for various pathogens, including *Plasmodium falciparum*, the malaria agent, through direct interaction with erythrocyte membrane protein 1 (PfEMP1) and reticulocyte-binding homolog protein 4 (PfRh4) (55, 62–66). The interaction with PfEMP1 causes red blood cells to become “sticky” and rigid, displaying the parasite phenotype known as “rosetting” or adhesion of infected erythrocytes to uninfected erythrocytes, which maintains infected red blood cells in the microvasculature, and avoiding destruction in the spleen and liver. At the structural level, rosetting depends on C3b-binding sites on LHR-B and LHR-C homologous repeats, even though it does not involve C3b. In contrast, PfRh4 interacts with CR1 CCP1 inhibiting the decay-accelerating activity without affecting C3b/C4b binding (55).

### 2.3 Complement receptor 2

Complement receptor 2 (CR2, CD21) is a type I membrane glycoprotein found on the cell surface of mature B cells, follicular dendritic cells, epithelial cells, and some T cells. CR2 contains 15-16 CCP domains depending on alternative splicing (67), which makes it the third largest CCP repeat protein within the RCA proteins after CR1 and FH. CR2 is the only RCA protein lacking complement regulatory functions; instead, CR2 links the innate and adaptive immune response during the activation of B cells through binding to its primary ligand, C3d, in a complex with CD19, CD81, and mIgM, which is thought to reduce the threshold of immune activation. Besides C3d and the C3d-containing opsonin iC3b (but not C3b) (68, 69), CR2 has three known ligands: IFNα (70, 71), the low-affinity IgE receptor CD23 (72), the glycoprotein gp350 of the Epstein-Barr virus (73, 74).

#### 2.3.1 CR2: a dynamic structure to bind opsonized surfaces

As sCR1 and FH, the structure of sCR2 has been studied by various structural and biophysical methods. The first images of sCR2’s elongated structure were obtained by electron microscopy (75). More detailed structural information has been obtained by a combination of SAXS and AUC (76). In solution, sCR2 has an *R*_G_ = 11.5 nm (*R*_G_/*R*_0 _= 4.1), *R*_XS-2 _= 1.2 nm (and no *R*_XS-1_), and a *D*_max_ = 38 nm (SAXS) and *s* ^0^_20,w_ = 4.2 nm (*f*/*f*_0 _= 2) (AUC). In contrast to sCR1 and FH, the CCP overall structural arrangement of sCR2 is more extended (although not fully extended) and only folds back partially onto itself (PDB ID 2GSX) (**Figure 1C**). Accordingly, the *D*_max_ derived from SAXS comes nearer to the theoretically maximum length of 54 nm. Another feature of CR2 concerns the length of the inter-CCP linkers, which is longer compared with CR1 and FH; in CR2, there are several inter-CCP linkers from four to eight amino-acid long. The greater length of the inter-CCP linkers gives CR2 a higher degree of flexibility, allowing it to reach a larger overall length than structurally similar proteins.

Isolated CR2 CCP domains have also been studied at the structural level. CCPs 1-2 have been shown to adopt a V-shaped structure by X-ray crystallography with implications for C3d/iC3b ligand binding (**Figure 1C**). Although the crystal structure of unbound CR2 CCPs 1-2 (PDB ID 1LY2) suggested a compact V-shaped arrangement with substantial flexibility at the junction between CCP1 and CCP2 domains (77), the solution NMR structures calculated for the same domains have shown a more open, but equally kinked, structure (PDB ID 1W2R) (78–80).

#### 2.3.2 Ligand binding at the interface between innate and adaptive immunity

The CR2 structure binds three relatively small protein ligands: C3d, gp350, and IFNα at CCPs 1-2, while a fourth ligand, CD23, binds both CCPs 1-2 and CCPs 5-8. The maximum dimensions of these ligands are 6.0 nm (C3d), 10.2 nm (gp350), 5.3 nm (IFNα), and 6.8 nm (CD23). Although CD23 is not much larger than C3d, CR2 uses two sets of CCP binding sites to latch onto it, a process likely favored by the flexibility of CR2. Therefore, CR2 can bind to antigen-C3d complexes on the B-cell surface and CD23 to bring the N-terminal tip of CR2 closer to membrane-bound IgE molecules on the B-cell surface (76).

The first crystal structure of CR2 CCPs 1-2 in complex with C3d showed a compact V-shaped structure where only CCP2 interacts with C3d (81). This was later disputed by constrained molecular modeling in 50 m*M* NaCl and mutagenesis data in 137 m*M* NaCl, which provided compelling evidence that both the CCP1 and CCP2 domains bind to the surface of C3d in a kinked conformation (PDB ID 1W2S) (79, 82). Later, a new crystal structure was published for CR2 CCPs 1-2:C3d, revealing a V-shaped conformation for CR2 CCPs 1-2 with a more extensive interface comprising residues from both CCP domains (PDB ID 3OED) (**Figure 1C**) (83). Although the conformation of CR2 CCPs 1-2 is similar in the two C3d complexes (RMSD 1.2-1.5 Å), the region of C3d involved in the interaction with CR2 CCPs 1-2 found in the crystal structure is more consistent with available functional and mutagenesis data.

The first structural glimpse into the CR2:C3d complex, indeed, into any large CCP-containing protein and its ligand, was obtained by SAXS and AUC (51). While sCR2 structure and oligomeric state remained unchanged in 50-137 m*M* NaCl, unbound C3d was shown to exist as monomers only in 137 m*M* NaCl; in 50 m*M* NaCl, C3d exists in monomer-dimer and monomer-trimer equilibria. Interestingly, the sCR2:C3d complex could be analyzed by AUC only in 50 m*M* NaCl, where the sedimentation coefficient shifted from 4.0 S (sCR2 alone) to 4.5 S (sCR2:C3d). The models put forward to rationalize the CR2:C3d complex (PDB ID 1W2R, PDB ID 1W2S) provide a solid foundation for future work, even if the details of the C3d binding interface may be better captured by the CR2 CCPs 1-2:C3d crystallographic structure (PDB ID 3OED) (76, 83).

Several features of the interaction between CR2 and C3d are worth remembering. Firstly, the extended, flexible, and relatively fold-back structure of CR2 and the V-shaped arrangement of CCPs 1-2 that is ideally positioned to interact with C3d-antigen complexes. Secondly, the constellation of weak and electrostatically modulated interactions between CR2 and C3d becomes physiologically significant only by avidity effects driven by receptor clustering. This weak summation of specific interactions appears discriminatory for B cells to respond only to antigens presented as multimeric C3d molecules clustered through surface-bound CR2 molecules. This parallels the weak CR1 interaction with C3b/C4b molecules on neutrophils, which is enhanced by the polymerization or multimerization of the ligand and receptor clustering. Other examples of this structural principle will be seen later.

#### 2.3.3 Implications of CR2 structure for diseases and immune evasion

CR2 was recognized as a receptor for the Epstein-Barr virus (EBV) very early on, mapping the CR2 binding region to the first two CCP domains and thereby in competition with C3d (68, 74). The gp350 protein on the surface of the viral membrane envelope interacts with CR2 during the first steps of EBV entry. Another virus that exploits CR2 as a receptor for viral entry is the human immunodeficiency virus 1 (HIV-1) (84). HIV-1 can infect B cell lymphocytes in a complement/C3-dependent and CD4-independent manner, thus facilitating viral dispersion and access to lymphoid organs (85). Besides viruses, the pathogenic yeast *Cryptococcus neoformans* has been shown to use an extracellular factor, the antiphagocytic protein 1 (App1), to bind to CR2 (and also CR3) and avoid complement-mediated phagocytosis by alveolar macrophages (86).

### 2.4 CR1 and CR2 are structurally selective receptors

CR1 and CR2 both recognize C3 activated fragments tethered to a biological surface by using a common structural framework, yet they accomplish a remarkable degree of selectivity through distinct binding modes and cell localization. As already seen, CR1’s main ligands are C3b, C4b, the AP/CP C3 convertases (monovalent binding sites), and the C5 convertases (bivalent binding sites), all markers of active complement opsonization. In contrast, CR2’s ligands are iC3b and C3d(g), both monovalent binding sites and markers of halted complement activation.

Although at face value CR1 and CR2 recognize distinct ligand sets, the fact that C3b, iC3b, and C3d(g) share the TED domain could potentially lead to overlapping binding sites. In fact, CR1 is known to bind iC3b and C3d with low affinity (87) in addition to the higher affinity binding to C3b/C4b. The solution to this apparent problem has been revealed by the X-ray crystal structures of CR1 CCPs 15-17:C3b (PDB ID 5FO9) (59) (**Figure 1B**) and CR2 CCPs 1-2:C3d (PDB ID 3OED) (83) (**Figure 1C**). By comparing these two complexes, which reflect the tightest receptor-ligand interactions (2 μ*M* for CR1 CCPs 15-17:C3b (59) and 22 n*M* for CR2 CCPs 1-2:C3d (78)), it is straightforward to see that the molecular surfaces recognized by either receptor are rather distinct. A small overlap is, however, created by CR1 CCPs 15-17 interacting weakly with the CUB-TED domains in C3b (59), interactions that are likely lost in iC3b. Furthermore, interaction of CR2 with C3d occurs through surfaces that are partially occluded in C3b, but sterically unimpeded in iC3b/C3d(g).

## 3 Integrin receptors

Integrins link the extracellular matrix (ECM) to the cellular cytoskeleton and associated signal transduction pathways and mediate cell-cell, cell-ECM, and cell-pathogen adhesion (88). Integrin-mediated interactions participate in cytoskeletal remodeling, phagocytosis, and cell migration (89). Complement receptors 3 (CR3) and 4 (CR4) belong to the integrin superfamily of type I transmembrane heterodimers (90). CR3 and CR4 are co-expressed in myeloid cells like neutrophil granulocytes, monocytes, macrophages, activated T and B lymphocytes, and lymphoid natural killer cells (91). They mediate immune adhesion-dependent processes such as adhesion to endothelium, phagocytosis of opsonized foreign particles, and other activation events that promote the innate and adaptive branches of the immune system (92).

These leukocyte-specific receptors bind multiple ligands like iC3b (93–95), ICAMs (96), fibrinogen (97), or lipopolysaccharide (LPS) (98). Despite their sequence homology, structural similarities, and overlapping ligands, CR3 and CR4 are functionally specialized, presenting a “division of labor” that makes them nonredundant receptors (34, 99).

Integrin signaling involves bidirectional communication between the extracellular environment and the intracellular cytoskeleton and signal transduction pathways, and the most accepted mechanism invokes an “inside-out” signaling precedence (100, 101). In the resting state, integrins adopt a bent, “closed”, ligand-free inactive state; signals initiated in the actin cytoskeleton can trigger a dramatic conformational change in the extracellular region of integrins, which become extended, “open”, ready to engage the ligand if available. When appropriate ligands are nearby, they can engage the receptors, initiating signaling processes from the “outside-in”.

### 3.1 The overall structure of the β_2_ integrins

Integrins are comprised of two noncovalently-associated protein chains: the α subunit (150-172 kDa) and the smaller β subunit (95 kDa), which is glycosylated. The cytoplasmic regions of integrins are very small compared to the large ectodomains. CR3 and CR4 share the same β_2_ chain (CD18) and belong to the β_2_ integrin family of adhesion receptors (90, 102), differing in their α chain, which is α_M_ in CR3 and α_X_ in CR4. The full CR3 heterodimer is also known as Mac-1 (Macrophage-1 antigen), CD11b/CD18, or integrin α_M_β_2_, and the CR4 heterodimer is also known as p150,95, CD11c/CD18, or integrin α_X_β_2_.

CD18/β_2_ integrins are restricted to leukocytes, and except for mast cells, which lose CD18 expression during differentiation, all leukocytes express one or more CD18 integrins (91). CR3 and CR4 have found utility in biomedicine as their expression in NK cells enables complement-dependent cytotoxicity toward anti-CD20 (rituximab)-coated cancer B cells, contributing to the treatment’s efficacy (103). CR3 and CR4 belong to the class of inserted (I) domain-carrying receptors. Fittingly, the N-terminal end of the α chain contains the iC3b-binding von Willebrand type A (VWA) domain or α chain inserted domain (αI), a specialized region characterized by a modified Rossman-fold architecture and a metal ion (Mg^2+^)-dependent adhesion site (MIDAS) motif (7). This relatively small domain is inserted between β-sheets (blades) 2 and 3 of the next domain in the α chain, a seven-bladed β-propeller domain, which allows the relative orientation of the αI and β-propeller to adjust flexibly (104). The aptly named Thigh, Calf-1, and Calf-2 domains complete the α chains. The α_M_ and α_X_ chains are homologous, with an overall sequence identity of ~60% (~47% in the αI domain). The functional discrimination of ligands by CR3 and CR4 is even more intriguing because most of the ligand recognition seems to be mediated by the 320-amino-acid αI domain, with the crucial involvement of the Mg^2+^ in the MIDAS. A comparison of the nature of known ligands suggests that strongly negatively charged molecules tend to be recognized by CR3, whereas CR4 tends to bind positively charged species (7). CR3 and CR4 are also homologous to two additional β_2_ integrins, the lymphocyte function-associated antigen 1 (LFA1, CD11a/CD18, α_L_β_2_) and α_D_β_2_ (CD11d/CD18).

The sequence of domains of the β_2_ chain from the N to the C terminus includes I-like, Hybrid, plexin-semaphorin-integrin (PSI), integrin epidermal growth factor (I-EGF) 1-4, and β tail (BT) domains. The β_2_-chain I-like domain interacts with the α-chain β-propeller domain to form a broad platform that supports the ligand-binding αI domain. The overall structure of the β_2_ chain domains contributing to the headpiece will be discussed later with available structural data for CR3 and CR4. As for the stalk region of the β_2_ chain, there is structural data for a substantial part, even if piecemeal.

The crystal structure of the PSI/Hybrid domain/I-EGF1 segment from the human integrin β_2_ chain was solved by X-ray crystallography at 1.8-Å resolution (PDB ID 1YUK) (105). The structure of this first part of the stalk revealed an elongated molecule with a rigid architecture stabilized by nine disulfide bonds. The PSI domain is wedged between the Hybrid and I-EGF1 domains, with extensive interfaces stabilized by contacts between conserved arginine and tryptophan residues. Soon after, there appeared two additional structures containing the PSI/Hybrid domain/I-EGF1 and additional I-EGF modules, I-EGF2 (PDB ID 2P26) and I-EGF2 and I-EGF3 (PDB ID 2P28) (106). The I-EGF is a cysteine-rich repeat module with a nosecone shape; four copies are located in the stalk region, where they relay activation signals to the ligand-binding headpiece. In the first structure, there was a prominent kink between the I-EGF1 and I-EGF2 modules, whereas, in the second structure, the three I-EGF modules adopted an extended conformation. The NMR structure for I-EGF3 and the NMR analysis of the interface contacts between I-EGF2 and I-EGF3 had been previously studied (PDB ID 1LY3) (107). The interdomain contacts between I-EGF domains 2 and 3 could be measured by NMR and were interpreted in terms of an approximate two-fold screw axis. In the NMR structure, the I-EGF domains 2 and 3 adopt an extended conformation connected by the “genu”, a highly flexible linker that allows extreme bending. Based on these data, the authors posited that the release of contacts of the headpiece with I-EGF modules 2 and 3 could trigger a switchblade-like opening motion springing the integrin into its extended, active conformation. Reanalysis of these structures in the context of the structure of the α_L_β_2_ headpiece in the closed conformation confirmed previous results while stressing the importance of proper disulfide pairing in the cysteine-rich I-EGF modules (PDB ID 5E6V, 5E6W, 5E6X) (108).

The structure of the single transmembrane helix of the β_2_ chain has been shown by NMR to use a membrane-snorkeling lysine residue (Lys702) to interact with acidic phospholipids in the membrane bilayer to stabilize the bent closed conformation (PDB ID 5ZAZ) (109). This interaction can be modulated by intracellular Ca^2+^, disrupting it and facilitating the acquisition of the extended open conformation. As this mechanism seems independent from the “inside-out” integrin signaling in T cell lymphocytes, it suggests a more prominent role for direct interactions of the β_2_ chain, membrane phospholipids, and Ca^2+^ in regulating integrin structure and conformational changes.

### 3.2 Complement receptor 3

**Figure 2:**
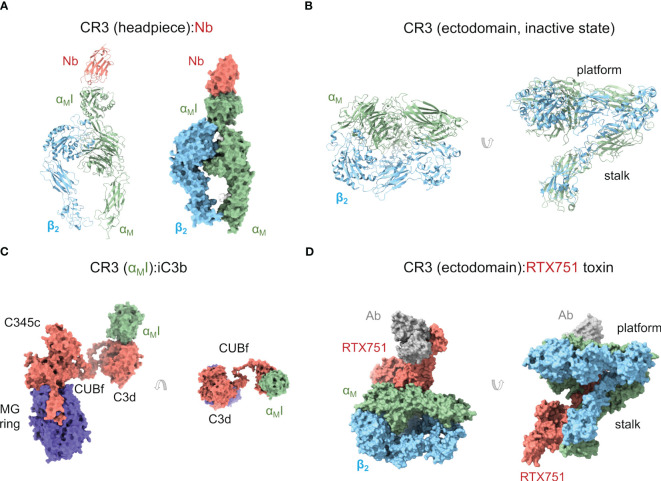
Complement receptor CR3. **(A)** Cartoon and molecular surface representations of the X-ray crystallographic structure of the CR3 headpiece in complex with a nanoantibody (Nb) (PDB ID 7P2D). The α_M_I domain is in the inactive, closed conformation. **(B)** Cartoon representation of the cryoelectron microscopy structure of the CR3 ectodomain (except for the α_M_I domain) in an inactive, closed conformation (PDB ID 7USM). **(C)** X-ray crystallographic structure of a complex between iC3b and CR3 α_M_I domain (PDB ID 7AKK) where iC3b adopts an extended conformation. The interaction between the TED/C3d domain of iC3b and CR3 α_M_I is shown in two orientations related by a 90° rotation. **(D)** Molecular surface representation of the cryoelectron microscopy structure of the CR3 ectodomain (except for the α_M_I domain) in complex with *B. pertussis* RTX751 toxin (PDB ID 7USL). In this structure, the CR3 ectodomain adopts a more extended conformation than in **(B)** through interactions with the toxin.

The initial work on the structure of CR3 was carried out by negative-staining electron microscopy on the headpiece (110) and by X-ray crystallography on the ligand-binding α_M_I domain, which has been most thoroughly characterized (111–113). More recently, the crystal structure of the CR3 headpiece (33) and the cryoelectron microscopy structure of the CR3 ectodomain (114) have advanced the field significantly (**Figure 2A, B**).

Crystal structures of the α_M_I domain in the open (PDB ID 1IDO) (112) and closed (PDB ID 1JLM) (113) conformations revealed the overall structure of the MIDAS motif and a ligand-binding regulatory mechanism whereby helix α_7_ plays a crucial role in the closed form by restricting access to negatively charged ligand residues. While in the open state a glutamic acid residue from a neighboring α_M_I domain completed the Mg^2+^ coordination sphere, in the closed form it was a key aspartic acid in helix α_7_ that played the role. Two other crystallographic structures of the α_M_I with the antagonist simvastatin (PDB ID 4XW2) (115) and with a ligand-mimicking antibody (PDB ID 3QA3) (116) have strengthened this idea by showing binding interfaces where the ligand contributes an acidic side chain to complete the metal coordination sphere.

CR3 and other β_2_ integrins show homotypic interactions or, at least, a tendency to form homotypic interactions. Tellingly, NS-EM studies on the CR3 headpiece showed large numbers of dimers (110). Even the α_M_I domain in the open conformation formed weak homodimeric interactions with crystallographic lattice neighboring molecules (112). Homotypic interactions may be desirable for β_2_ integrins as they must accommodate large concentrations during receptor clustering or result from the loose ligand specificity. The first structure of a β_2_ integrin with a complement factor ligand was that of CR3 α_M_I in complex with C3d (PDB ID 4M76), the main ligand for CR3 (111). This structure revealed the binding mode of C3d to α_M_I, characterized by an aspartate side chain of C3d chelating the MIDAS motif of α_M_I, which was occupied by a non-physiologic Ni^2+^ cation from the crystallization condition. Although the buried surface area is relatively small, the interaction is strong enough (affinity (*K*_D_) is in the micromolar range) to stabilize the complex and is comparable in area and affinity to other integrin complexes. A key insight from this structure showed that the C3d surface engaged by α_M_I is masked in C3b by a well-folded CUB domain, effectively ruling out the possibility of an α_M_I interaction with C3b. In iC3b, however, the cleavage by FI inside the CUB domain causes it to unfold, making the α_M_I binding motif accessible.

Another fascinating insight from the α_M_I:C3d structure was its compatibility with the binding of C3d by CR2 through its CCPs 1-2 domains (7). This hypothetical α_M_I:C3d:CR2 complex was interpreted as a hand-over or transfer of C3d-opsonized antigens from CR3-bearing macrophages to CR2-bearing B lymphocytes. This process might act like an MHC-independent antigen presentation mechanism bridging the innate and adaptive branches of immunity. It would be interesting to characterize the hand-over in more cellular structural detail as the involvement of three distinct surfaces (macrophages, B cells, and opsonized particles) renders the entire process challenging.

A recent structure by our group has shown a complex between the entire iC3b and the α_M_I domain (PDB ID 7AKK) (**Figure 2C**) (34). In this structure, the MIDAS motif of α_M_I was fully charged with Mg^2+^. The interaction between α_M_I and the TED domain of iC3b was identical to the interaction previously observed between α_M_I and C3d (111). More interestingly, the structure of CR3 α_M_I:iC3b revealed two potential interfaces for the α_M_I domain on the MG ring of the C3c fragment. This is relevant because the C3c moiety has been known to contribute to CR3 binding in interaction assays (111) and, perhaps more importantly, because iC3b-opsonized surfaces are phagocytosed by CR3-expressing macrophages at much lower opsonin concentration than C3d-opsonized surfaces (117), begging the question about what the role of C3c in CR3 binding might be. In one of these α_M_I:C3c interfaces involving C3c MG1-2 domains, the C3c moiety of iC3b would conserve an “original” orientation with respect to the surface-anchored TED domain (“upright”), while in the other one, involving the C3c MG3-4 domains, the C3c moiety would be required to turn around and lie “upside-down” with respect to the TED domain. Surface plasmon resonance (SPR) binding experiments and site-directed mutagenesis of interfacial residues on the α_M_I domain indicated that both interfaces may be relevant *in vivo*. Additional evidence will be necessary to clarify the physiological role of the C3c moiety of iC3b for CR3 recognition. Meanwhile, an enticing hypothesis is that iC3b behaves as a modular platform comprising a surface-anchored C3d and a more detached C3c moiety that collaborate to bind the CR3 ectodomain in the highly concentrated environment of the cell-particle interface. A modular iC3b would facilitate binding by increasing the number of low-affinity contacts (avidity effect), restricting the angular spread of CR3:C3d complexes to increase CR3:iC3b packing efficiency (alignment effect), and making it possible for C3d(g) and C3c moieties from the same or different iC3b molecules to collaborate in CR3 binding.

Although the role of CR3 as a complement receptor is well-attested, the capacity of CR3 (and CR4) to recognize and bind to a wide variety of other ligands remains puzzling. CR3, for example, interacts with many macromolecular components of the coagulation system (e.g., fibrinogen, fibrin, kininogen, plasminogen, heparin), denatured proteins, and oxidative and degrative products of lipids and glycans (7). Analysis of the electrostatic properties of the ligand-facing molecular surface has shown that it is a markedly negatively charged surface, which is in line with the preference of CR3 for cationic ligands like the intrinsically unstructured myelin basic protein (MBP) (118) and the antimicrobial peptide LL-37 (119), as long as they also have a carboxylate moiety. These properties have been used to justify including CR3 and CR4 as scavenging receptors, i.e., those receptors like CD36 and the receptor for advanced glycation end products (RAGE) that enable cellular removal of decayed macromolecules in extracellular space (7). With its preference for cationic ligands that can interact with cell membranes, CR3 could function as a receptor for clearing cellular debris associated with membrane damage. CR3’s multitude of ligands and the fact that its outside-in signaling dampens inflammatory responses are in line with this proposal; CR4, instead, lacks anti-inflammatory signaling, suggesting that it may be functionally distinct from CR3 in this context (7).

More recently, the crystal structure of the CR3 headpiece has been determined in complex with a nanoantibody at 3.2-Å resolution (PDB ID 7P2D) (33) (**Figure 2A**). This structure provides the first high-resolution structure of CR3 beyond the α_M_I domain. In this structure, the CR3 headpiece adopts the closed conformation, which resembles those of LFA1 and CR4 in the same state. The nanoantibody used for crystallization could sterically block C3d binding in an Mg^2+^-independent manner but, surprisingly, acted as an agonist for cell-bound α_M_β_2_, thus apparently increasing affinity for the iC3b ligand. These seemingly contradictory observations could be reconciled by proposing that additional conformational flexibility on the integrin and iC3b might permit interactions in cell surface-bound integrin that are not observed in solution or binding experiments *in vitro*. Indeed, the current understanding of both α_M_β_2_ and iC3b structures suggests that they can interact in complex, heterogeneous environments using multiple domains in the crowded cell surface environment (32, 34).

Within a few months of the publication of the previous structure, the cryoelectron microscopy structure of the CR3 ectodomain at 2.7-Å resolution was published (PDB ID 7USM), along with the structure of a complex with an adenylate cyclase toxin RTX751 from *Bordetella pertussis* and a stabilizing Fab (PDB ID 7USL) (114) (**Figure 2D**). While the unbound α_M_β_2_ adopts the closed conformation seen for the CR3 headpiece and closed CR4 ectodomain (see below), RTX751-bound α_M_β_2_ was able to achieve a more extended conformation through stabilizing interactions with RTX751, closely resembling the conformations of extended α_X_β_2_ ectodomains (120, 121). The closed and extended conformations of CR3 were related by a hinge motion of the headpiece relative to the tailpiece, pivoting between the Thigh/β-propeller in the α_M_ headpiece with the Calf-2 domain in the tailpiece. The observation of a partially extended conformation in the RTX751 complex suggests that CR3 must be able to spontaneously sample more extended conformations by built-in flexibility around the headpiece-tailpiece hinge angle, which is consistent with previous observations (107).

### 3.3 Complement receptor 4

Earlier structural work on the CR4 headpiece by NS-EM showed an overall structure like that of other integrins, particularly very similar to that of CR3 (120). Maintaining the inactive state of the α_X_I domain requires the correct pairing of the α_X_ and β_2_ chains, as shown by several experimental approaches (e.g., 122). When CR4 adopts an extended conformation, an α-helix in the I-like domain exerts a pull on the α_X_I domain that opens it up for binding.

The α_X_I domain was the first αI domain to be elucidated by X-ray crystallography (121). The crystallographic structure of the α_X_I shows a similar overall fold to that of CR3. Studies of CR4 revealed the flexible connection of the α_X_I domain with the β-propeller domain, suggesting that rotational freedom was required for efficient ligand binding (108). This property might also be necessary for binding structurally diverse ligands since the chelation of acidic ligand groups by the core cation in the MIDAS motif is likely to restrict ligand movement to pivoting around the Mg^2+^ cation.

NS-EM micrographs of the CR4 headpiece bound to iC3b revealed up to two independent CR4 α_X_I binding sites on the iC3b MG ring (110). The dominant site, present in all complexes, was located close to the macroglobulin domains MG3-4, and a secondary site, less frequently occupied, was found near the C345c domain. Interestingly, the two α_X_I binding sites do not overlap with the MG binding sites identified for α_M_I (34), suggesting that the two receptors saturate all potential binding sites on the MG ring of iC3b without directly competing. This does not imply the potential for simultaneous binding of iC3b by CR4 and CR3, which would be sterically impeded, but it shows that the αI domains have some degree of selectivity in ligand binding.

A structural chemical feature of CR4 α_X_I is the presence of a “ridge” of positively charged residues on the ligand-facing molecular surface (7). The electrostatic properties of α_X_I are markedly distinct from those of the homologous α_M_I and α_L_I domains (7, 34). This property has been invoked to explain why CR4 can selectively bind polyanionic molecules more efficiently than CR3 (7). Indeed, highly negatively charged polymers/molecules like heparin, nucleic acids, LPS, and osteopontin are proficient ligands for CR4. In parallel to the potential role of CR3 as a scavenger receptor for polycationic species, a receptor like CR4 with a strong preference for polyanionic species could serve complementary functions as a scavenger receptor by clearing excessive amounts of negatively charged proteins, detecting the presence of the negatively charged bacterial cell wall and LPS-rich membranes, and potentially alerting the immune system about their presence.

**Figure 3:**
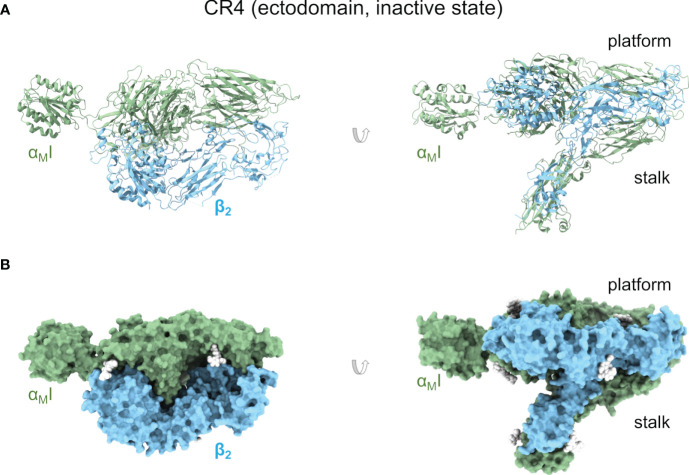
Complement receptor CR4. **(A)** Cartoon representation of an X-ray crystallographic structure of the CR4 ectodomain in an inactive but metastable structure that anticipates activation (PDB ID 4NEH). Two orientations related by a 90° rotation are shown. **(B)** As in **(A)** but as a molecular surface representation. Native glycan moieties are shown as white spheres.

The structure of the complete CR4 ectodomain has been solved by X-ray crystallography in a bent, resting state (PDB ID 3K71, 3K72, 3K6S, 5ES4) (121) and a metastable, transition state (PDB ID 4NEN, 4NEH) (120) (**Figures 3A, B**). In the resting structure, the α_X_I domain is in the inactive conformation, as expected for the ectodomain’s inactive state. Surprisingly, the α_X_I domain shows a high degree of flexibility around the loops connecting it to the β-propeller domain. This suggests a more dynamic coupling between the αI domain with implications for the allosteric transmission of information along the integrin’s body. The second set of structures of the α_X_β_2_ ectodomain represents an intermediate or transition state from the bent, closed, to the open, extended conformations, with a crystal lattice contact stabilizing the α_X_I domain in an open conformation. A key feature of this structure consists in the unwinding of much of α_X_ α_7_ helix and its insertion into the interface between the β-propeller and the βI domains. The elevation (lift-off) of the α_X_I domain above the headpiece’s platform facilitates large-scale extensional and rotational motions of sufficient amplitude to communicate allosteric changes across the length of CR4.

## 4 Complement receptor of the immunoglobulin family

The complement receptor of the immunoglobulin (Ig) family (CRIg) is a type I transmembrane Ig superfamily member first cloned during a search for homologs of the junctional adhesion molecule (JAM) family (123). CRIg is also known as V-set and immunoglobulin domain-containing protein 4 (VSIG4), Z39Ig, UNQ317, and PRO362. In humans, there are three spliced isoforms of CRIg. The most common allelic form contains 399 amino acids and is known as huCRIg(L), whereas isoforms 2 and 3 are shorter, with 274 and 296 amino acids, respectively. Isoform 3 is also known as huCRIg(S). Full-length CRIg contains a signal peptide, a V-type Ig-like 1 domain, a C2-type Ig-like 2 domain, several potential *O*-glycosylation sites, and an intracellular domain with two potential phosphorylation sites, and is structurally related to the B7 family of immune regulatory proteins (124). Whereas huCRIg(L) comprises both Ig-like domains, huCRIg(S) contains only the V-type Ig-like 1 domain.

CRIg is expressed in tissue-resident and sinusoidal macrophages like the Kupffer cells, the resident macrophages in the liver, and it mediates phagocytosis of particles opsonized by any of its two ligands, C3b and iC3b (125). Besides stimulating pathogen opsonophagocytosis, CRIg is also known to be a potent inhibitor of the activation of the C3 convertase of the alternative pathway by binding to C3b, acting as a negative regulator of complement activation. In contrast to the other C3 fragment receptors (CR1 to CR4), CRIg is found on a constitutive recycling pool of membrane vesicles where it participates in the internalization of C3-opsonized particles from the bloodstream by Kupffer cells. Instead, CR1, CR3, and CR4 are located on secretory vesicles that fuse with the plasma membrane upon cytokine stimulation of the cells and internalize ligands through a macropinocytotic process only after receptor cross-linking (126). Besides its localization in the liver, CRIg is also abundantly expressed in resident macrophages from several fetal and adult tissues, with the highest expression attained in the lung and placenta. The main function of CRIg is to act as a potent negative regulator of T-cell proliferation and IL-2 production (127).

### 4.1 CRIg has two consecutive Ig-like domains

**Figure 4:**
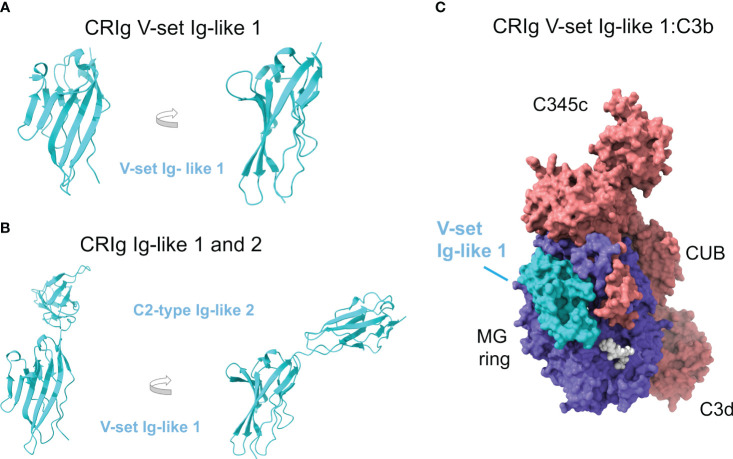
Complement receptor CRIg consists of two Ig-like domains and binds the β-chain of C3b. **(A)** Crystallographic structure of the V-set Ig-like 1 domain of CRIg, in cartoon representation and two orientations (PDB ID 2ICC). **(B)** AlphaFold predicts with high confidence the structural model of the C2-type Ig-like 2 domain of CRIg (AlphaFold AF-Q9Y279-F1). The consecutive Ig-like 1 and 2 domains are shown in cartoon representation and two orientations; the orientation of the V-set Ig-like 1 matches panel A for comparison. **(C)** Structure of CRIg V-set Ig-like 1:C3b (PDB ID 2ICF) in molecular surface representation. C3b is colored according to chain (the α chain in red, the β chain in blue), and CRIg V-set Ig-like 1 domain is in cyan. Native glycan chains in C3b are shown in white spheres.).

CRIg contains two Ig domains known as Ig-like 1 and Ig-like 2 domains. The crystallographic structure of Ig-like 1 (residues 19-137) was determined to 1.2-Å resolution, showing a V-set Ig-like fold that resembles the antibody variable domain, responsible for providing the binding specificity (PDB ID 2ICC) (128) (**Figure 4A**). Subsequent crystal structures of the same V-set Ig-like 1 domain from human and mouse have been determined in complex with a nanoantibody that blocks binding with C3c and C3b (PDB ID 5IMK, 5IML, 5IMM, 5IMO) (129).

In contrast, the structure of Ig-like 2 (residues 143-226) has not been solved, although sequence homology has identified its fold as a C2-type Ig-like domain reminiscent of constant antibody domains that provide the effector functions. The AlphaFold model corresponding to full-length CRIg contains the predicted structure of the Ig-like 2 domain (AlphaFold AF-Q9Y279-F1) with a very high degree of confidence (**Figure 4B**).

The Ig-like domain consists of antiparallel β-strands arranged into two sheets linked by a disulfide bond. V-set domains can be distinguished from C2-type domains because they show the variability associated with antigen/ligand recognition, and the domain is longer with two extra strands tucked into the middle of the domain (C’ and C’’). The first β-sheet in Ig-like 1 comprises β-strands A’, G, F, C, C’, and C’’, and the second one of β-strands B, E, and D (**Figures 3A, B**).

### 4.2 CRIg binds to the β-chain of C3b and iC3b

C3b and iC3b are the main ligands of CRIg. Other C3 proteolytic fragments have been tested for CRIg binding, including C3, C3a, and C3d, and other homologous complement factors like C4 and C5, all with negative results (125).

The structures of CRIg Ig-like 1 domain bound to C3c (PDB ID 2ICE) and C3b (PDB ID 2ICF) (**Figure 4C**) have been determined by X-ray crystallography at 3.1-Å and 4.1-Å resolution, respectively (128). Since C3d is not a ligand for CRIg, the C3c moiety shared by C3b/iC3b becomes the most likely binding site for CRIg Ig-like 1 domain. Furthermore, C3c and C3b differ essentially in the lost TED domain (C3dg) while they share most of their structural core with minimal deviation: ~0.6 Å root mean square distance over 479 Cαs of the α-chain and 642 Cαs of the β-chain.

CRIg Ig-like 1 domain binds to C3c or C3b identically without restructuring or inducing conformational changes in the ligand. Residues from β-strands A’, F, G, C’, and C’’ from one of the β-sheets and β-strand B from the second β-sheet are engaged in the interaction, with β-strands C’ and C’’ contributing most of the interactions. The hairpin loop between β-strands C’ and C’’ sticks into the cavity in the center of the keyring-shaped β-chain of C3b.

The binding site is quite large (2,670 Å^2^ of solvent-accessible surface). It overlaps the cavity inside the C3c macroglobulin ring, crossing it diagonally and ending at the interfaces between consecutive MG domains MG3-MG4 and MG5-MG6 (**Figure 3C**). This binding site places CRIg on the opposite side of the surface-anchored C3dg/TED domain, a sterically accessible location facilitating binding. The most extensive contributions to binding are made by MG3 and MG6, supplemented by MG4, MG5, and LNK. Compared to C3, in C3c and C3b MG3 has rotated by 15° and there is a movement of the helical section in the LNK region, which appear to be necessary to form the CRIg binding site and thereby explain why CRIg cannot bind to C3.

The V-set Ig-like 1 domain of CRIg is sufficient for high-affinity binding to C3b/iC3b, even though this domain alone binds iC3b more strongly than to C3b. Although the presence of the C2-type Ig-like 2 domain is not required for binding, it restores high-affinity binding to C3b to the same level observed for iC3b (125). Interestingly, dimeric C3b (C3b_2_) (130) is bound more tightly to CRIg than monomeric C3b, a relevant result because multimeric C3b is supposed to represent the physiologic state of C3b on opsonized surfaces.

Significantly, CRIg binding to C3b inhibits the C3 and C5 convertases of the alternative pathway since CRIg blocks the generation of C3a and C3b by C3 convertase and C5b by C5 convertase. In a series of elegant experiments, site-directed mutations introduced in CRIg residues in contact with C3b MG3, MG5, or MG6 at the center, periphery, or outside the CRIg:C3b interface led to a strong, weak, or negligible effect on CRIg-mediated inhibition of the C3 convertase, evaluated in hemolytic assays with rabbit red blood cells (128). This proved that CRIg inhibition of the C3 convertase depended on its interaction with C3b.

In contrast to other complement negative regulators, CRIg does not inhibit the alternative pathway convertases by dissociating the catalytic subunit Bb (decay accelerating activity) or promoting C3b degradation by FI (cofactor activity). In contrast, it appears that CRIg, once bound to the β-chain of C3b, can sterically hamper association with the C3 and C5 alternative pathway convertases. Remarkably, CRIg fails to inhibit the C3 and C5 classical pathway convertases, indicating that the functional effect is confined to C3b and the alternative pathway.

### 4.3 Implications for diseases and immune evasion

CRIg is an essential receptor for the clearance of complement-opsonized particles, which are recognized and phagocytosed by Kupffer cells in the liver. Pathogens and immune complexes are shuttled in the circulation by CR1-bearing erythrocytes and handed over to CRIg-expressing Kupffer cells in the liver in a dynamic process relying on immune adherence that prevents systemic inflammation and immune complex diseases associated with aberrant vascular deposition (8). This extremely efficient mechanism of blood-borne pathogen clearance can catch Gram-positive and Gram-negative bacteria (8) and eukaryotic parasites (131).

## 5 Anaphylatoxin G-protein coupled receptors

The anaphylatoxins C5a and C3a, generated by the proteolytic activation of C5 and C3, respectively, are potent chemoattractants and pro-inflammatory mediators. The cognate receptors of C5a are C5aR1 (C5aR/CD88) and C5aR2 (C5L2/GPR77), and the cognate receptor of C3a is C3aR (C3aR). The analogous “anaphylatoxin” released by proteolytic activation of the C3-homologous zymogen C4, C4a, has thus far defied a functional description and, perhaps significantly, lacks a specific receptor (132, 133).

C5aR1/C5aR2 and C3aR belong to the G-protein coupled receptor (GPCR) family and, more precisely, to the rhodopsin family and class A, two equivalent groupings at the highest hierarchy level of the two main GPCR ordering systems (134–136). Based on sequence homology and functional similarity of GPCR, the three receptors are classified into the complement peptide group inside class A (135). In contrast, within the rhodopsin family defined by the phylogenetic classification of human GPCR, C5aR1/C5aR2 are members of the chemokine cluster of γ-group, while C3aR belongs to the purine receptor cluster of δ-group (134).

The three anaphylatoxin receptors share structural features common to all GPCR. They are comprised of an extracellular N-terminal region, seven transmembrane α-helices (TM1-7), connected sequentially by intracellular (ICL) or extracellular (ECL) loops, and an intracellular C-terminal region (137–139). To refer to TM residues, we will follow the Ballesteros-Weinstein numbering (140), and for the remaining residues, we will use the name of the containing motif (ECL, ICL, N-ter, or C-ter) as superscripts.

The anaphylatoxin receptors are widely expressed in immune cells from the myeloid and lymphoid lineages and nonimmune cells like epithelial cells and neurons (141). By binding their cognate ligands, the receptors are implicated in diverse cellular functions, physiological processes, and pathologies, mainly related to the immunological system (141–148).

The clinical relevance of these receptors in different acute and chronic disorders, mainly with an inflammatory etiology, has triggered a great interest in developing specific and effective modulators (149). In this context, an exhaustive investigation has aimed to reveal insights into the structure-function of these receptors to increase the understanding of how they carry out their functions and to support the finding of modulators with clinical potential.

### 5.1 Complement receptor 5a 1

#### 5.1.1 Structure of C5aR1

**Figure 5:**
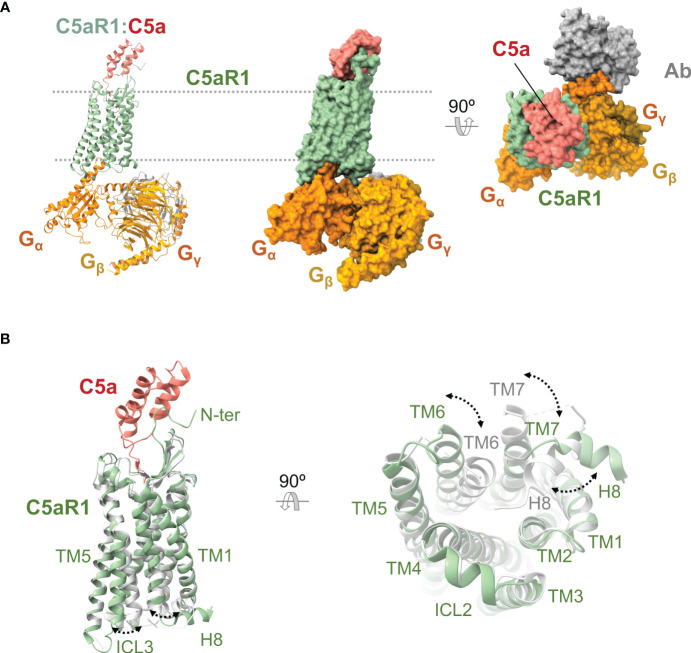
Anaphylatoxin receptor C5aR1 in complex with C5a and conformational changes underlying activation. **(A)** Cryoelectron microscopy structure of C5aR1:C5a bound to heterotrimeric G protein comprising the Gα, Gβ, and Gγ subunits (shown in orange, yellow, and gold), and a stabilizing antibody (shown in gray) (PDB ID 7I65). C5aR1 is shown in green, and C5a in red. The same view is shown of the cartoon and molecular surface representations of the complex. We show a 90° rotated view of the complex on the right in molecular surface representation. **(B)** Superposition of the active conformation of the C5aR1:C5a complex (PDB ID 7I65) (C5aR1 in green, C5a in red) and an inactive reference structure (PDB ID 6C1Q) (C5aR1 in grey; the antagonist PMX53 is not shown for clarity). Side (left) and bottom (right) views are shown. Conformational changes are indicated by dashed lines between and labeling of structural elements that occupy distinct positions in the active *versus* inactive conformations.

C5aR1 was discovered in 1978 (150), and the coding sequence of its 350 amino acids was cloned and determined in 1991 (151, 152). While the unliganded receptor has defied structural determination, recently published high-resolution X-ray crystal (PDB ID 5O9H, 6C1Q, 6C1R) and cryoelectron microscopy (PDB ID 7Y64, 7Y65, 7Y66, 7Y67) structures (**Figure 5A**) have provided insights into the structural features of this receptor.

The structure of the core transmembrane region is conserved with other class A GPCR (138, 139, 153) including the overall helical arrangement and kinks, the placement of TM3 at the center of C5aR1 with a lower tilt-angle with respect to the plane of the lipid bilayer, W^6.48^ (W255^6.48^) near P^6.50^ (P257^6.50^), the PIF motif (P214^5.50^, I124^3.40^, F251^6.44^), an intrahelical sodium coordination site (D82^2.50^, N292^7.45^, N296^7.49^) at the cytoplasmic side, the NPXXY motif (N296^7.49^, P297^7.50^, I298^7.51^, I299^7.52^, Y300^7.53^) and the DRY/F motif (D133^3.49^, R134^3.50^, and F135^3.51^). On the extracellular side, C5aR1 presents a conserved disulfide bond (C109^3.25^ and C188^ECL2^) and a β-hairpin conformation in ECL2 that resembles other peptide binding receptors (138, 154, 155).

At the intracellular region, the ICL2 exhibits a two-turn α-helical structure, and some C-terminal residues form the conserved eighth amphipathic α-helix (H8) of three turns long (35, 138, 154, 155). C5aR1 can oligomerize *in vivo*, forming homodimers and perhaps higher-order homo-oligomers (156, 157) and heterodimers with CCR5 (158) and C5aR2 (159).

Other post-translational modifications observed in C5aR1 are an *N*-glycosylation at N5 (160), tyrosine sulfation at Y11 and Y14 (161) at the N terminus, and serine phosphorylation at S314, S317, S327, S332, S334, and S338 in the C terminus (162); albeit to a lesser degree, threonine residues can also be phosphorylated (158).

#### 5.1.2 C5a-binding orthosteric site

The cognate ligands of C5aR1 are the anaphylatoxin C5a and its dearginated product C5a^desArg^, which have been extensively researched (163, 164). Other ligands of the C5aR1 have been recently discovered (165).

The high-resolution structures of liganded C5aR1 have revealed an orthosteric and an allosteric binding site (35, 36, 154, 155). The orthosteric binding site consists of an interhelical solvent-exposed amphipathic cavity located at the extracellular side of the receptor (35, 36, 155) (**Figure 5A**). This site can be divided into a polar region and a hydrophobic cage. The polar region spans from the outer edge to one side of the binding site and comprises hydrophilic and charged residues recruited from TM3-7 and the membrane-proximal part of ECL2. The hydrophobic cage is mainly formed by hydrophobic residues from TM1-3, TM7, and ECL1, which extend from one side to the bottom of the binding site. The orthosteric site can bind peptide ligands of up to eight residues (amino acid positions numbered P1-P8) with various pharmacological effects, including C5a, BM213, C5a^pep^, PMX53, and C089. Water molecules can be involved in ligand binding, forming a polar network connecting the ligand to residues from the polar surface (155). The allosteric binding site is an extra-helical hydrophobic cleft formed by residues from the middle regions of TM3, TM4, and TM5, which bind non-peptide ligands such as NDT9513727 or Avacopan through shape complementarity, hydrophobic interactions, and a key hydrogen bond with W213^5.49^ (154, 155). The N terminus and the turn region of the ECL2 can also participate in binding orthosteric ligands (35, 36).

The structures of C5aR1 in complex with C5a and the G_i_ protein show that C5a binds in a C-terminus-inside mode, and the receptor interacts with three distinct regions of the anaphylatoxin (35) (**Figure 5A**). Firstly, an amphipathic stretch of the receptor’s N terminus (L22-D27) interacts with a cavity between C5a α-helices H2 and H4 and the H3-H4 loop. Secondly, the orthosteric site accommodates the last eight residues of C5a. Inside the orthosteric site, the C5a C terminus adopts a hook-shaped conformation, establishing strong interactions with residues at the polar surface while accommodating inside the hydrophobic cage. In this orientation, the side chain of C5a L^P6^ interacts with a triad of receptor residues denominated IWI region (I91^2.59^, W102^ECL1^, I116^3.32^) (35, 36). Finally, the amphipathic turn region of ECL2 (E180^ECL2^-P183^ECL2^) makes contact with C5a in a cleft assembled by α-helices H1, H2, and H4 and the H2-H3 loop.

Other C5aR1 ligand peptides occupy the orthosteric binding site in a similar fashion to the C5a C terminus (35). The main differences in binding mode between C5a and these peptides have been observed in the hydrophobic cage of the orthosteric site, particularly in the interactions established between the peptides’ P6 and P7 positions and the IWI region and surrounding residues (e.g., L92^2.60^, S95^2.63^, H100^ECL1^, P113^3.29^, and V286^7.39^) (35, 155). A unique feature of the cyclic peptide PMX53 is a hydrogen bond between W^P5^ and P113^3.29^ (155).

The recent flurry of C5aR1 structures has made important contributions to understanding the structure-function relationship of C5aR1 and how C5a binds and activates the receptor. However, a few questions remain unanswered. For example, receptor aspartic acid residues upstream of L22 (166, 167) and the sulfated Y11 and Y14 (161) had been shown to play important roles in C5a binding. Still, current structures have not revealed any direct interactions between these residues and the ligand. Therefore, how these residues contribute to C5a binding has not been explained. They may establish direct interactions with C5a (168) or be required for the structural conformation of the binding site (169).

The recent structural evidence agrees with a two binding sites model previously proposed (170). In this model, the first binding event with C5a engages a site 1 in C5aR1 composed of the N terminus and the ECL2, which directly contacts the helical core of C5a; this binding event favors a conformational change of C5a that promotes binding to the site 2, composed of the orthosteric pocket, which interacts with C5a C terminus. In support of this model, kinetic and thermodynamic analyses by atomic force microscopy of the binding interaction have confirmed that both sites 1 and 2 contribute to the high-affinity binding of C5aR1:C5a through a cooperative mechanism (171). The markedly different conformation adopted by bound C5a with respect to the free form lends further support to this model. In the bound conformation, C5a shows a rearrangement of the C terminus and the helical bundle, which changes from a 1.5-turn helix form placed near the helical bundle (as seen in free C5a) to an unfolded conformation in bound C5a (35). These changes have been rationalized as a bi-directional information flow between C5a and C5aR1 (166). According to this view, the first binding event would be characterized by C5aR1 site 1 promoting or favoring a conformational change in C5a, and the second binding event in a fully extended conformation of C5a inside site 2 would then activate the receptor.

Orthosteric ligands vary widely in their binding affinity for C5aR1. While C5a interacts with C5aR1 with a dissociation constant *K*_D_ = 1-5 n*M* (151, 167, 172, 173), its dearginated version C5a^desArg^, which only differs from C5a in the last amino acid, R74 (174), has a 10 to 100-fold lower binding affinity. However, peptides derived from the C-terminal end of C5a show modest binding affinities, suggesting that the engagement of the receptor N terminus and the ligand coupling to the orthosteric site (35, 175) are important features for the strong binding of C5a. The lower affinity of C5aR1 for C5a^desArg^ could be explained by the putative lack of interactions between R74 at P8 in the orthosteric site (35).

#### 5.1.3 C5a-mediated activation of C5aR1

C5aR1 is activated by orthosteric agonists (170, 175–177), which cause rearrangements in the extracellular, transmembrane, and intracellular regions of the receptor (35, 36) (**Figure 5B**), leading to a conformation in which it can trigger intracellular signaling pathways by recruiting transducers (138, 139, 153). At the extracellular region, the N terminus appears not to be involved in the C5aR1 conformational changes since this stretch is not required for receptor activity (161, 166, 175, 177). The ECL1 and especially its WXFG motif perform a critical role in C5aR1 activation (178). The ECL2 accomplishes a striking function in ligand-mediated activation of C5aR1 beyond its role in ligand binding (35, 179). In contrast to ECL1 and ECL2, ECL3 seems not to be involved in C5aR1 activation (178). Other residues essential for C5aR1 activation were found at the core of the transmembrane helix bundle and inter-helical interfaces (178, 180, 181).

In line with the common mechanism of class A GPCR (153, 182), an activation mechanism has been proposed for C5aR1 based on the receptor conformational changes observed between an active state (bound to C5a and G_i_) and an inactive state (bound to PMX53 and Avacopan), and the currently available receptor structure-function data (35, 36, 155). Initially, the coupling of C5a into the orthosteric site causes TM displacements that loosen a hydrophobic zipper formed by I116^3.32^, M120^3.36^, W255^6.48^, and Y290^7.43^ at the extracellular side of C5aR1. This zipper is proposed to act as a lock tethering TM3, TM6, and TM7 in an inactive configuration, and movements key for loosening it are an upward rotation of the extracellular region of TM3 and a TM6 downward displacement, which may be caused by the engagement of C5a L^P6^ by the IWI region and the R^P8^-Y258^6.51^ interaction, respectively. Next, TM shifts keep taking place disturbing the PIF motif at the hydrophobic core. Sequentially, the reorganization of the helical bundle core cause conformational changes at the cytoplasmic side of C5aR1 that include a pronounced outward displacement of the intracellular section of TM6, an H8 swing from its location between TM1 and TM7 oriented toward the receptor’s center to a classic conformation near to the lipid bilayer, alterations of NPXXY and DRY/F motifs, the sodium coordination site collapse, and the opening of an interhelical water-accessible cavity (**Figure 5B**). Eventually, the rearrangements promote receptor-transducer interactions and, ultimately, intracellular signaling (35, 36).

Other remarkable conformational changes associated with C5aR1 activation by C5a include an uncommon placement of Y300^7.53^ that may be important for the particular H8 reorientation, TM3 and TM7 distancing their extracellular sides and approaching their intracellular segments, and rearrangements of clusters of water molecules located into the interhelical cavities that are separated by W255^6.48^ and I116^3.32^ at the helical core of the receptor (35, 36, 155).

#### 5.1.4 Modulation and selectivity of C5aR1 activity

C5aR1 exhibits functional selectivity as it triggers distinct downstream signaling and cellular responses in a ligand and cell-type-dependent fashion. The ability of C5aR1 to activate different transducers and the variety of mechanisms by which its activity can be modulated contribute to this functional selectivity. Agonist-activated C5aR1 can signal through heterotrimeric guanine nucleotide-binding proteins (G-proteins) with α-subunits of the G_i/o_ and Gα_16_ families (183–187). Coupling the Gα_i1_ subunit involves C5aR1 residues from ICL2, ICL3, TM3, TM5, and TM6, and Gα_i1_ residues from the β_2_-β_3_ loop, β_6_ strand, and α_5_ helix. Remarkably, the α_5_ helix of Gα_i1_ inserts into the receptor intracellular cavity taking up part of the space filled by H8 in the inactive C5aR1 (35, 36). The receptor segments involved in G-protein signaling include the DRY/F and NPXXY motifs, particularly the ICL3 (180, 188, 189).

The G-protein specificity of C5aR1 seems to lie in the three ICLs and particularly the receptor C terminus, which could work together to restrict the receptor interaction with G-proteins (188). Noteworthy, C5aR1 can bind G_i_ even in the absence of agonist stimulation, suggesting that, although G-protein pre-coupling to C5aR1 is not essential for ligand binding, the pre-coupling could increase the receptor affinity for ligands as C5a:C5aR1 binding fosters the G-protein coupling to the receptor (190–192).

The main phosphorylation sites identified in C5aR1 include serine (S314, S317, S327, S332, S334, and S338) and threonine residues at the C terminus (158, 162, 193). A likely PKC phosphorylation site has also been identified in the ICL3 (194). C5aR1 phosphorylation seems hierarchical as the initial modification of S332/S334 or S334/S338 is required for the efficient phosphorylation of the remaining sites (195). Several kinases, including GRK (GRK2, GRK3, GRK5, and GRK6) and PKC (PKCβ), can be implicated in C5aR1 phosphorylation (193, 196, 197). GRK2 can be translocated to the plasma membrane in response to C5a, and the receptor ICL3 is the intracellular stretch preferentially bound by GRK2 and PKCβII (198). Receptor phosphorylation is involved in β-arrestin recruitment (158, 193, 199), receptor internalization (158, 194, 199, 200), and desensitization (193, 195). Although the precise roles of these phosphorylation sites have not yet been elucidated, one or several of the C-terminal serine residues (S327, S332, S334, and S338) appear to be the most relevant phosphorylation sites for β-arrestin recruitment and C5aR1 internalization and desensitization (193). L318^H8^ plays an important role in the receptor conformation that can be efficiently phosphorylated and internalized (201).

Activated C5aR1 can recruit β-arrestins 1 and 2 (199). GRK 2, 3, 5, and 6 have all been shown to facilitate the recruitment of β-arrestins 1 and 2 to C5aR1 (187, 202). β-arrestins 1 and 2 recruitment by C5aR1 may be followed by receptor internalization and downstream signaling (199, 203). The C terminus of C5aR1 is required for efficient homologous receptor internalization (194). C5aR1 internalization has been observed as a clathrin-dependent process that is associated with the β-arrestin recruitment by the receptor. C5aR1 internalized in early endosomes can be targeted to lysosomes for degradation or recycled to the cell membrane. The trafficking of C5aR1 through these pathways appears to depend on the cell type (199–201). Recycling of C5aR1 likely requires its dephosphorylation by several phosphatases, including protein phosphatase 1 (PP1) (193, 200). Although a structure of C5aR1 binding to β-arrestins is lacking, impaired β-arrestin binding by different phosphorylation-deficient C5aR1 mutants indicates that a stable association between C5aR1 and β-arrestin likely requires a certain degree of receptor phosphorylation (158, 193, 199). C5aR1 can also couple with the Wiskott-Aldrich syndrome protein (WASP), which binds to the receptor’s C terminus (204).

Agonist-induced and unliganded C5aR1 receptor oligomerization have been observed (205). Oligomerization likely plays important functional or regulatory roles *in vivo*, but little is known about how C5aR1 assembles into oligomers and the implications for receptor activity. Structurally, TM1, 2, and 4 may be involved in C5aR1 dimerization (157, 181), while neither the N nor the C terminus is required for C5aR1 dimerization (205). Only in one of the solved structures with the extra-helical antagonist NDT9513727 (PDB ID 5O9H), C5aR1 has been observed in a non-crystallographic homodimeric organization, although this arrangement might not be physiologically relevant (154, 155); structural comparison with the homodimer of the smoothened (SMO) receptor (206) has lent support to the C5aR1 homodimer arrangement. Remarkably, the agonist activation of C5aR1 homodimers and phosphorylation of only a monomer can lead to the internalization of these dimers (205).

C5aR1 activity can also be modulated by antagonists and inverse agonists that bind to its orthosteric or allosteric site (154, 155). These allosteric modulators trap the receptor in the inactive state by stabilizing the network of hydrophobic interactions that maintain the receptor in the inactive state or by sterically hindering the helical rearrangements required for receptor activation. The structures of PMX53:C5aR1 (PDB ID 6C1Q, 6C1R) reveal how the cyclic peptide PMX53 sits in the orthosteric site, interacting with residues of the polar surface that interact with C5a and inserting the side chains of the d-cyclohexyl alanine (dCha^P4^) and W^P5^ in the vicinity to the IWI region (155). In the allosteric site, NDT9513727 (PDB ID 6C1Q) and Avacopan (PDB ID 6C1R) establish interactions with the hydrophobic core of the transmembrane region, including the PIF motif (155).

Several C5aR1 agonists display ligand bias, i.e., differences in receptor signaling via G-protein signaling and β-arrestin recruitment (35, 207, 208). Ligand bias may depend on interactions between the agonist and ECL2 and between the ligand P6 and the IWI region, which are important for C5a-mediated β-arrestin recruitment. Interestingly, C5aR1 inhibitors display ligand bias effects: while PMX53 preferentially inhibits G-protein coupling over β-arrestin recruitment, Avacopan shows the opposite trend (35).

### 5.2 Complement receptor 5a 2

#### 5.2.1 Structural features of C5aR2

The gene encoding human C5aR2 (*HsC5L2*) was cloned and sequenced many years after *HsC5aR1* (209), and what its specific functions may be is still an active research area. In particular, the structure of C5aR2 has not been elucidated experimentally. As C5aR1, C5aR2 belongs to the class A GPCR superfamily and therefore shares the overall structure of the model GPCR. Although the sequence identity and similarity with C5aR1 are relatively high (~36.4% and 57.1%, 128 identical and 73 similar residues), the difference is sufficiently extensive to make us cautious about extrapolating structural facts about C5aR1 onto C5aL2 without critical assessment.

The following putative and potential structural features have been recognized by sequence homology. Firstly, the seven transmembrane helical core that is a hallmark of all GPCR. Secondly, the N terminus contains an *N*-glycosylation site at N3 (143). Thirdly, the presence of a disulfide bond between C107^3.25^-C186^ECL2^. Fourthly, serine and threonine phosphorylation sites in the ICL3 and C terminus. And, finally, a remarkably shorter ICL3 compared with C5aR1 and other related GPCR.

As far as differences are concerned, C5aR2 lacks a canonical DRY/F motif at the end of TM3, which is substituted for by the sequence D131^3.49^, L132^3.50^, and C133^3.51^, thereby resulting in the obligate uncoupling of C5aR2 from heterotrimeric G proteins (210). It also lacks an S/T-X-R/K phosphorylation site in ICL3. Finally, the NPXXY motif in TM7 lacks the tyrosine residue, which has been replaced by F291^7.53^ (209, 211).

#### 5.2.2 C5aR2 binds C5a and C5a^desArg^

C5aR2 has two high-affinity ligands: C5a and C5a^desArg^. Like C5aR1, C5aR2 exhibits a higher affinity for C5a over C5a^desArg^. While the affinity for C5a is similar for C5aR1 and C5aR2, the affinity of C5aR2 for C5a^desArg^ is one order of magnitude tighter than C5aR1 (210, 211). This, together with the slower dissociation rates of C5a from C5aR2 (210), has motivated the proposal that the primary physiological role of C5aR2 may be modulating the activation through C5aR1.

C5aR2 does not seem to share the exact binding mechanism for orthosteric ligands proposed for C5aR1. Indeed, C5a and C5a^desArg^ display distinct binding modes to C5aR2. The C5aR2 N terminus plays crucial roles in C5a^desArg^ binding, especially through the acidic and tyrosine residues and the tyrosine sulfation posttranslational modification (212).

Underscoring this differential recognition of C5a by C5aR1 and C5aR2, two partial agonists (P32 and P59) that are selective for C5aR2 over C5aR1/C3aR have been discovered (213). Conversely, the well-characterized C5aR1 antagonist PMX53 is perfectly selective toward C5aR1 and biased toward G_i_ signaling and does not bind C5aR2 (159).

Besides the two cognate ligands, there has been some controversy over the possibility that C3a, C3a^desArg^ (ASP), and C4a could also bind to C5aR2 (211). Although enticing, this proposal has not been supported by recent data. None of the three proteins activates the receptor (211, 214); it seems none binds to it (210, 213).

#### 5.2.3 C5aR2: a C5a receptor that recruits β-arrestin

There are striking differences between C5aR2 and C5aR1 concerning localization, phosphorylation, internalization, and regulation.

To begin with, C5aR2 is mainly localized inside the cell rather than in the plasma membrane and does not show a rapid internalization in response to C5a binding (210, 215). The internalization mechanism of C5aR2 appears to be different from that of C5aR1 and is only clathrin-dependent. In fact, internalized C5aR2 can also be constitutively recycled to the plasma membrane by a clathrin-dependent process (215). These processes suggest that C5aR2-bearing cells can uptake C5a and C5a^desArg^ and either keep them intracellularly, target them for degradation, or release them back into the extracellular environment. Since C5aR2 exhibits a greater efficiency in ligand uptake and processing than C5aR1, C5aR2 can be posited as the main receptor involved in internalizing, retaining, and degrading C5a in natively expressing C5aR2 cells (215).

Regarding phosphorylation status, C5aR2 is phosphorylated to a lesser degree than C5aR1 in response to C5a binding (210). C5aR2 can recruit β-arrestin 1 and 2 in an agonist-dependent manner (187, 213, 214), a process involving phosphorylation sites introduced by GRKs 5-6 (187). Besides C5a-mediated recruitment, C5aR2 can also pre-couple both β-arrestins in the absence of agonist (187, 216). The direct interaction of C5aR2 with β-arrestin 1 has been shown by NS-EM (187). Interestingly, both β-arrestins exhibit different conformations bound to C5aR2 than those bound to C5aR1 (187). In contrast with C5aR1, C5aR2 showed a greater β-arrestin 2 recruitment stimulated by C5a/C5a^desArg^ that was equally strong for the two anaphylatoxins (214, 216).

An intriguing aspect of C5a receptors is that C5aR2 can form heterodimers with C5aR1 constitutively and also in an agonist-dependent manner (159). It has been hypothesized that through heterodimerization, C5aR2 might regulate C5aR1 activity and cooperate functionally (159, 216).

C5aR2 does not signal through the heterotrimeric G proteins (187, 210), and grafting the C5aR1 motifs that engage with G proteins such as the DRY/F motif, ICL3, and the NPXXY motif does not complement C5aR2 (215). The inability of C5aR2 to couple with G proteins explains that C5aR2 plays a much smaller role in intracellular signaling than C5aR1 (210, 214).

Based on the lack of G-protein signaling, clearing of extracellular C5a/C5a^desArg^, and β-arrestin recruitment and internalization properties, C5aR2 has been proposed to be a recycling decoy receptor (215). Although this is an attractive theory, C5aR2 still contributes to intracellular signaling processes in a variety of roles, including modulation of the phosphorylation state of several transducers (e.g., p90RSK and ERK1/2) and intracellular calcium mobilization (187, 217). Different functions attributed to C5aR2 consist in the modulation of other immunological receptors, like C5aR1, C3aR, chemokine-like receptor 1 (CMKLR1), or different pattern recognition receptors (PRRs) (217). Through these functions, C5aR2 is involved in regulating complex cellular responses, such as releasing cytokines from macrophages (213, 217).

### 5.3 Complement receptor 3a

#### 5.3.1 Structure of C3aR

Like the other anaphylatoxin receptors, C3aR was classified as a GPCR when the encoding gene was cloned and its primary sequence determined (218, 219). Analysis of the primary sequence revealed a distinctive structural feature of C3aR: an unusually long ECL2 of about 172 amino acids, predicted to be highly flexible, with a disulfide bond formed between C95^3.25^ and C172^ECL2^. In contrast, its N terminus is shorter than that of C5aR1.

C3aR has several posttranslational modifications, including *N*- and *O*-glycosylations and tyrosine sulfations (220). S266^ECL2^ is *O*-glycosylated (221), and N9^N-ter^ and N194^ECL2^ have been predicted to be *N*-glycosylated. Y174, Y184, Y188, Y317, and Y318 in the ECL2 can carry sulfations. The ICL3 and C terminus of C3aR contain serine and threonine residues that can be phosphorylated (197).

**Figure 6:**
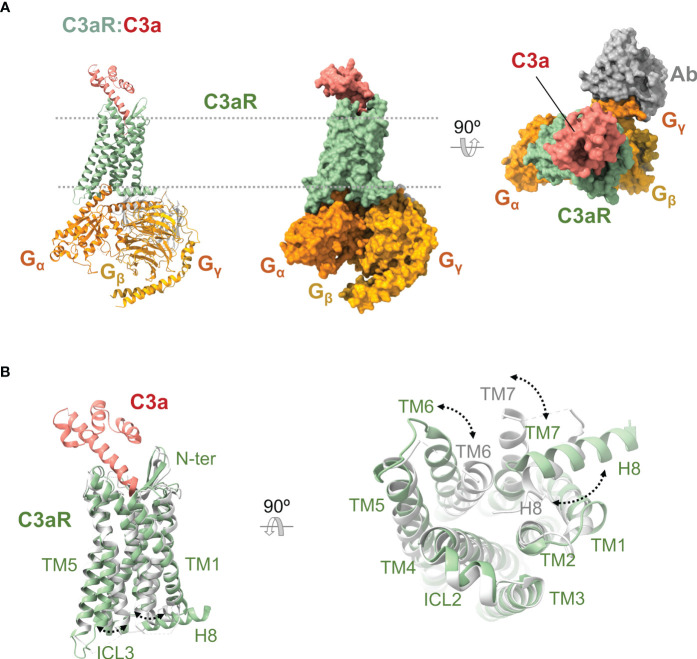
Anaphylatoxin receptor C3aR in complex with C3a and conformational changes underlying activation. **(A)** Cryoelectron microscopy structure of C3aR:C3a bound to heterotrimeric G protein comprising the Gα, Gβ, and Gγ subunits (shown in orange, yellow, and gold), and a stabilizing antibody (shown in gray) (PDB ID 8HZ2). C3aR is shown in green, and C3a in red. The same view is shown of the cartoon and molecular surface representations of the complex. We show a 90° rotated view of the complex on the right in molecular surface representation. **(B)** Superposition of the active conformation of the C3aR:C3a complex (PDB ID 8HZ2) (C3aR in green, C3a in red) and an inactive reference structure (PDB ID 6C1Q) (C5aR1 in grey; the antagonist PMX53 is not shown for clarity). Side (left) and bottom (right) views are shown. Conformational changes are indicated by dashed lines between and labeling of structural elements that occupy distinct positions in the active *versus* inactive conformations.

The cryoelectron microscopy structures of C3a-free (PDB ID 8HK3) and C3a-bound C3aR coupled to a G_i_ heterotrimeric protein (PDB ID 8HK2) (**Figure 6A**) have only recently become available, representing a striking leap ahead in the field (36). As suspected, the long ECL2 loop is highly flexible, and only the first 16 residues (V159-K175) were resolved, adopting a β-hairpin conformation.

#### 5.3.2 C3a last five amino acids are critical for C3aR interaction

The cognate ligand of C3aR is the C3a anaphylatoxin, which binds with high affinity (~1 n*M*) (218, 219, 222). Compared to C5aR1, C3aR does not bind C3a^desArg^ (223). C3a recognition by C3aR has been proposed to follow a mechanism like that of C5aR1 with some differences. Like C5aR1, a two binding site model has been proposed (36, 222).

The primary or effector binding site (orthosteric site) consists of an interhelical amphipathic pocket found in the extracellular region of the receptor and formed by residues from TM3, TM5-7, and ECL2 (**Figure 6A**). The orthosteric site of C3aR is smaller than that of C5aR1, and it can only accommodate the last five residues of C3a (P1-P5). The C-terminal end of C3a inside the binding site adopts a hook-shaped conformation enabling multiple interactions with the amino acids in the cavity, including ionic and hydrophobic interactions and hydrogen bonds. Remarkably, R77 of C3a establishes some of the critical interactions, which include a salt bridge with D417^7.35^, a cation-π interaction with Y393^6.51,^ and two hydrogen bonds with Y174^ECL2^ and R340^5.42^ (36).

The secondary binding site encompasses residues from the outermost segments of ECL2 and ECL3 that still make contact with the α_4_ helix of C3a. Even though this secondary binding site plays a less important role in engaging C3a, some functionally relevant contacts have been found in this region, e.g., the salt bridge between C3aR D404^ECL3^ and C3a R65 (36).

While the C3aR N terminus has shown little implication in C3a binding (36, 222, 224), its ECL2 plays an important role in the anaphylatoxin coupling. Site-directed mutagenesis studies have established that aspartic acid residues along the N-terminal and C-terminal ends of ECL2 (D183, D186, D325, D326, and D327) and sulfation of Y174 are required for high-affinity binding of C3a (36, 220, 222). Besides mediating C3a binding, the ECL2 appears vital for arranging the helical bundle functionally (224).

The selectivity toward their cognate anaphylatoxin ligands of C3aR and C5aR1 is in part due to the amino acid composition of the C terminal tails; in particular, C3a G74 seems to be essential for C3aR selectivity (36). Furthermore, the N terminus of these receptors also has shown a significant contribution to this selectivity, so the amino terminus of C3aR appears to hinder C5a binding (224). This is in accordance with the composition and implication of these N termini in the anaphylatoxin binding.

#### 5.3.3 Activation of C3aR

Once liganded, C3aR can couple and signal by different heterotrimeric G proteins with α subunits from the G_i/o_ and G_q_ families (164). C3aR recognizes residues in the β_2_-β_3_ loop, β_6_ strand, and α_5_ helix of the Gα_i_ subunit through residues from TM3-6, ICL2, and ICL3. The coupling to the heterotrimeric G_i_ to C3aR is similar to C5aR1, although the α_5_ helix of the Gα_i_ subunit is inserted more deeply, TM6 is displaced by ~1.5 Å, and the ICL3 displays a distinct topology and broader interaction with Gα_i_ (36) (**Figure 6B**).

C3a stimulation leads to C3aR phosphorylation on Ser and Thr residues in a dose-dependent manner by GRKs 2/3/5/6 and PKC (197). Removal of the C3a stimulus is followed by C3aR dephosphorylation. GRKs have shown different functions over C3aR signaling in mast cells. While GRK2/3 are involved in agonist-induced desensitization, GRK5/6 are implicated in cell degranulation (225). Phosphorylation at Ser459^C-ter^, Thr463^C-ter^, Ser465^C-ter^, Thr466^C-ter^, and Ser470^C-ter^ is involved in β-arrestin 2 recruitment at C3aR and receptor desensitization in mast cells (226). Thr463^C-ter^, Ser465^C-ter^, Thr466^C-ter^, and Ser470^C-ter^ have important roles in C3aR internalization, which occurs in an agonist-dependent manner (227). Agonist-induced phosphorylation of C3aR performs an important role in signal transduction from the receptor. In C3a-stimulated mast cells, phosphorylation is required for CCL2 production, but these modifications seem to attenuate a degranulation response (228).

C3aR activity can be modulated by β-arrestins 1 and 2. β-arrestins can inhibit C3a-induced ERK1/2 phosphorylation and perform other regulatory activities. For example, in mast cells, β-arrestin 2 is involved in C3aR desensitization, internalization, and inhibition of the C3a-induced NF-kB activation and CCL4 generation; β-arrestin 1 contributes mainly to cell degranulation (229).

## 6 Concluding remarks

The progressive recognition of the complement system as a driver of inflammatory and autoimmune diseases (11, 27, 28, 30, 31) has incentivized the functional and structural study of the complement system and, in particular, of the complement receptors, their ligands, and their complexes.

Work over many years has established the existence of at least four structurally distinct classes of complement receptors: the CCP/SCR mosaic receptors CR1 and CR2 (along with the negative regulators MCP and DAF), the Ig superfamily receptor CRIg, the β_2_ (CD18) integrin receptors CR3 and CR4, and the anaphylatoxin GPCR receptors C5aR1, C5aR2, and C3aR. This diversity likely reflects the enormous span of evolutionary time over which cellular immunity has coevolved with the complement system (230–232).

Although the diversity of functions carried out by the complement system and immune cells defies classification, specific unifying themes can be recognized.

Firstly, CCP/SCR mosaic receptors (CR1 and CR2) are long and flexible molecules that can survey considerable distances both in the plane of the membrane and above it to find their main ligands: C3b/C4b and C3 and C5 convertases (CR1) and C3d and iC3b (CR2). CR1 downregulates complement activation through the well-known decay-accelerating and cofactor activities, also exerted by the negative regulators MCP and DAF (membrane-bound) and FH and C4BP (fluid phase). In contrast, CR2 lacks complement regulatory activities but it has a more specialized role in bridging innate immunity with adaptive immunity by handing over complement-opsonized pathogens from macrophages to antigen-presenting cells in the spleen and other lymphoid organs.

Secondly, CRIg recognizes C3b (and iC3b) but does it with a structurally unrelated fold (Ig-like) and targets the β-chain of the proteolytically activated C3 fragments. Its unique binding mode is used by the tissue-resident macrophages that express it (mostly, Kupffer cells in the liver) to snatch complement-opsonized pathogens and cellular debris and facilitate their clearance by phagocytosis.

Thirdly, the β_2_ integrin receptors CR3 and CR4 have played an important role in establishing the structural basis for activation (e.g., the transition from a bent, closed, inactive conformation to an extended, open, active state) and ligand recognition by integrin receptors. Besides binding to iC3b (and to C3d in the case of CR3), these receptors have remarkable ligand promiscuity, employing a small but versatile domain (the αI domain) to recognize them. Their study has highlighted the degree to which surface concentration effects (the so-called 2D concentration, through avidity, compartmentalization, and clustering) and diversity in the structural presentation of ligands can influence the outcome of their interaction.

Fourthly, the anaphylatoxin GPCR receptors have aroused considerable interest as C5a and C3a have been associated with inflammatory diseases, and agonists/antagonists are available. Although the first structures of C5aR1 were obtained by X-ray crystallography, cryoelectron microscopy has allowed the determination of high-resolution structures of C5aR1:C5a and C3aR:C3a complexes within a short time. This acceleration promises a revolution in the structural understanding of anaphylatoxin receptors, how anaphylatoxins are recognized, and how binding triggers receptor activation and signal transduction. The detailed structural knowledge of receptor:anaphylatoxin complexes will also allow better agonists and antagonists to be engineered and validated as therapeutics.

The applications of the structural inquiry of the complement receptors and their ligands are important. Knowledge about the structural organization of the receptors and the ligand complexes advances a fundamental understanding of the immune system. It often results in unexpected and fascinating results, like the proposal that CR2 can hand over iC3b/C3d-opsonized surfaces from macrophages to antigen-presenting cells to stimulate (prime) adaptive immunity. More pragmatically, the structures of ligand complexes often make it possible to elucidate the mechanism of action of known (or suspected) receptor agonists and antagonists, with implications for developing more efficient treatments. It also paves the way for the rational or semi-rational discovery of new agonists and antagonists, increasing their utility, selectivity, and safety. For example, discovering new allosteric inhibitors/antagonists of the anaphylatoxin receptors could result in new treatments.

What are the challenges for the future regarding the structural biology of complement receptors?

The structural catalog of complement receptors is still incomplete. Although state-of-the-art protein structure prediction methods like AlphaFold (233) are filling in the gaps with enticing structural hypotheses (e.g., the C2-type domain of CRIg), it is important to elucidate the structures of receptors, ligands, ligand complexes, and complexes involving them and other immune system components and the host-pathogen machinery. In connection with this central endeavor, developing new methods for protein production and the characterization of protein-protein and protein-ligand complexes will continue to play a significant role as enabling technologies (234, 235).

Much remains to be elucidated regarding structural diversity and modularity, especially in 2D surfaces, highly concentrated clusters of receptors and ligands, and cell-cell interactions. The role of cryoelectron microscopy in advancing the field of structural biology of transmembrane receptors cannot be denied, and the trend is likely to strengthen further (236). A goal for the future should be to integrate cellular structural biology approaches (237) to gain insights into how complement receptors (and complement regulators, membrane-bound or otherwise) function in the physiological and pathological context.

As extracellular pathogens have evolved a seemingly endless repertoire of complement-evasive factors (238, 239), intracellular pathogens have evolved molecular mechanisms to hijack all the non-GPCR complement receptors, MCP, and DAF to gain entry to the cell (240, 241). Strategies aimed at restricting pathogen entry into their host cells targeting the virulence factors or their cognate complement receptors would greatly benefit from a complete understanding of the structural determinants of the interaction.

Finally, these advances should also result in a more subtle and precise appreciation of the differences between the various animal models in use in the field of complement. Applying structural methods and tools to the complement factors and complement receptors of mice, rats, and other animal models beside humans will allow a more informed understanding of the differences in the complement biology between these animals, with important implications for developing disease models and therapies.

## Author contributions

FJF and MCV conceived the study. JS-L wrote the first draft of the sections on anaphylatoxin receptors. KdlP wrote the first draft of the sections on complement integrin receptors. FJF and MCV wrote the sections on CR1, CR2, and CRIg with support from KdlP. FJF and MCV wrote the final version of the manuscript. All authors contributed to manuscript revision, read, and approved the submitted version.
